# A secular trend of increasing pubertal BMI change among Swedish adolescents

**DOI:** 10.1038/s41366-021-01011-0

**Published:** 2021-11-06

**Authors:** Maria Bygdell, Claes Ohlsson, Jenny M. Kindblom

**Affiliations:** 1 Centre of Bone and Arthritis Research, Department of Internal Medicine and Clinical Nutrition, Institute of Medicine, Sahlgrenska Academy, University of Gothenburg, Gothenburg, Sweden; 2 Department of Drug Treatment, Sahlgrenska University Hospital, Region Västra Götaland, Gothenburg, Sweden; 3 Pediatric Clinical Research Center, Sahlgrenska University Hospital, Region Västra Götaland, Gothenburg, Sweden

**Keywords:** Paediatrics, Epidemiology, Epidemiology

## Abstract

Pubertal BMI change is an independent risk marker of cardiovascular mortality/morbidity. Previous studies demonstrated a secular trend of increased childhood BMI but it is unknown if there is a concomitant secular trend regarding pubertal BMI change. The aim of this study was to describe the trend in pubertal BMI change. We collected heights and weights before and after puberty from school health records and military conscript records for boys born every five years during 1946–1991 (*n* = 3650, total cohort) and calculated pubertal BMI change (young adult BMI at 20 years of age minus childhood BMI at 8 years of age) for all study participants. A secular trend of increasing pubertal BMI change during the study period was observed. The increase in pubertal BMI change (0.27 kg/m^2^ per decade [0.22; 0.32]) explained 54% of the secular trend of increasing young adult BMI (0.50 kg/m^2^ per decade [0.43; 0.57]). We made the novel observation that there is a secular trend of increasing pubertal BMI change. We propose that the secular trend of increasing pubertal BMI change might contribute more than the secular trend of increasing childhood BMI to the adverse cardiovascular health consequences associated with the ongoing obesity epidemic.

## Introduction

An elevated BMI in adulthood is one of the leading risk factors for cardiovascular mortality and morbidity and various other conditions [1]. High BMI during childhood has also been suggested to be a risk factor for several diseases in adulthood (cardiovascular diseases, cancer, and diabetes) [2–4]. The global obesity epidemic has increasingly affected adults as well as children during the last three decades. We and others have shown that both mean BMI and the prevalence of obesity during childhood have increased [5, 6], and a dramatic increase in young adult BMI has been observed [7, 8].

We recently made the pivotal observation that pubertal BMI change (BMI at 20 years of age minus BMI at 8 years of age) correlates only marginally with childhood BMI, indicating that these two developmental BMI variables have the potential to contribute separate information as risk markers for adult disease. This notion is supported by our recent findings that excessive pubertal BMI increase, but not childhood BMI, is an independent risk factor for cardiovascular mortality, heart failure, and stroke [9–12]. In addition, we have recently shown that pubertal BMI change is associated with coronary artery calcification score, not mediated by midlife BMI or major cardiovascular risk factors, and with acute coronary events [12]. Moreover, pubertal BMI change, but not prepubertal childhood BMI, is associated with the amount of visceral fat in young adult men [13]. Despite that pubertal BMI change is an independent risk marker of cardiovascular mortality and morbidity [9–11, 14], the trend over time for this developmental BMI variable is unknown.

Prevention of overweight and obesity during development and in extension, future illness, is a societal and parental responsibility and the preventive measures differ depending on the age of the child [15]. In order to understand how to target prevention, it is important to characterize the temporal patterns for the increase in BMI during development. The aim of the present study was, therefore, to describe the trend in pubertal BMI change over a time span of 45 years.

## Methods

The population-based BEST Gothenburg cohort includes individuals that completed school in Gothenburg municipality and afterwards had their school health record stored in the central archive. The school health records include data on height and weight from regular health visits at child healthcare centers and school healthcare throughout childhood until the children finished secondary school. These health visits include all children (>98.5% of all children in the municipality for school healthcare from the calendar year 1952 [16]). The present BEST Gothenburg sub-cohort includes boys born consecutively from January 1^st^ and onwards every five years between 1946 and 1991. We also collected the height and weight of the included participants from mandatory military conscription tests. Conscription was mandatory until 2010 for all Swedish men. The inclusion criteria for this study were to have at least one measurement of childhood BMI and at least one measurement of young adult BMI. Individuals were excluded if a personal identity number (PIN) was missing. The study has been approved by the local ethics committee at the University of Gothenburg.

In Sweden, a PIN is assigned to every citizen at birth or immigration. Using the individuals’ PIN, the best cohort was linked with the Longitudinal Integration Database for Health Insurance and Labor Market Studies at Statistics Sweden, and the country of birth for every study participant and their parents were retrieved. Country of birth was categorized as Sweden if the study participant and both parents were born in Sweden, or as other for other countries or missing information.

The BMI variables were calculated as previously described [9]. For these calculations, R (standard packages) were used, for all other statistical analyses SPSS (version 24) were used. Descriptive statistics for the BMI variables were calculated for every birth cohort. The overall trend was analyzed using a linear regression model adjusted for country of birth, and the comparison between mean BMI for the different birth cohorts was tested using one-way analysis of variance (ANOVA) followed by Dunnett’s post hoc test.

## Results

**Fig. 1:**
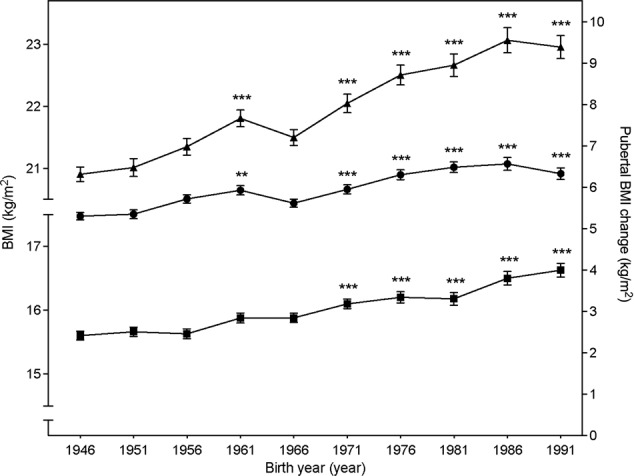
Mean pubertal BMI change (filled circles, right y-axis), childhood BMI (filled squares, left y-axis), and young adult BMI (filled triangles, left y-axis) for boys included in the BEST Gothenburg cohort born 1946–1991. The data for childhood BMI are part of our previously published results [5]. Values are presented as mean ± SEM. Statistically significant differences compared with birth cohort 1946 are indicated: **p < 0.01, ***p < 0.001. BEST = BMI Epidemiology Study; BMI = Body Mass Index; SEM = Standard Error of the Mean.

**Fig. 2:**
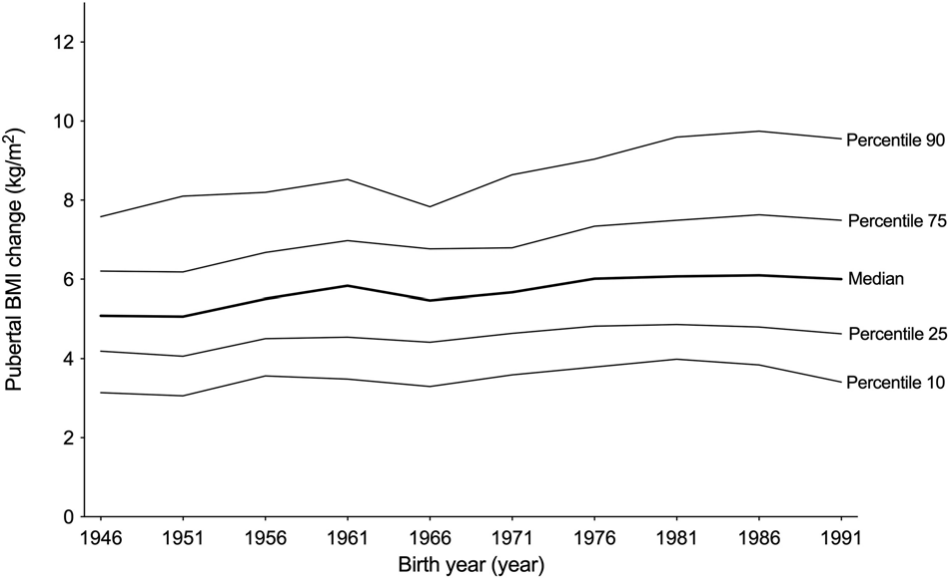
Distribution of percentiles of pubertal BMI change for boys included in the BEST Gothenburg cohort born 1946–1991. BEST = BMI Epidemiology Study; BMI = Body Mass Index.

In the present study, 10 birth cohorts of boys born every five years during 1946–1991 were included (*n* = 365 each, total *n* = 3650). The mean (SD) pubertal BMI change was 6.0 (2.3) kg/m^2^ for the entire cohort (Supplemental Table 1). We observed a clear increase in pubertal BMI change during the study period (Fig. 1). The distribution of pubertal BMI change is displayed in Fig. 2 and shows that pubertal BMI change has increased across percentiles. A linear regression model demonstrated that for every decade later birth year during the period 1946–1991, an increase of 0.12 SD of pubertal BMI change (95% CI 0.10;0.14) was observed. The association was linear (p for quadratic term = 0.24). To account for a possible secular trend in height, we adjusted our analyses for height at 8 years and final height. Neither adjustment for height at 8 years, nor for final height, substantially changed our results (0.11 SD increase in pubertal BMI change per decade [0.09;0.14] adjusted for height at 8, and 0.12 [0.10;0.15] SD increase in pubertal BMI change per decade, adjusted for final height).

As previously observed, we also observed increases in childhood BMI and young adult BMI in the study cohort 1946–1991 (Fig. 1). We next evaluated the relative contribution of trends of increased childhood BMI and of increased pubertal BMI change to the secular trend of increased young adult BMI during the study period. Young adult BMI at 20 years increased by 0.50 kg/m^2^ (0.43; 0.57) per decade during the study period. This increase in young adult BMI was reflected by the concomitant increases in pubertal BMI change (0.27 kg/m^2^ per decade [0.22; 0.32]) and childhood BMI (0.23 kg/m^2^ per decade [0.19; 0.27]). Thus, the increase in pubertal BMI change explained 54% of the secular trend of increased young adult BMI during the study period.

To be able to investigate whether the results presented here are confounded by changes in the composition of the study population during the long time span this study covers, we performed a sub-analysis according to country of birth for the study participants and their parents. All analyses were therefore replicated using a sub-cohort of boys born in Sweden and with parents born in Sweden (*n* = 2856). The results did not differ notably from the main analyses using the total cohort (data not shown).

## Discussion

We have previously demonstrated that excessive pubertal BMI increase is a novel risk marker for several cardiometabolic diseases, but the secular trend of this BMI variable is unknown. In the present population-based cohort study, we made the novel observation that there is a secular trend of increased pubertal BMI change.

During the last decades, an obesity epidemic both among adults and young adults has emerged worldwide [7]. One study shows that mean BMI at age 18 years has increased 7%, and the prevalence of overweight and obesity have increased 2.4 and 3.5 times, respectively, using BMI data from the military conscription register including Swedish men born 1971–1995 [8]. Children are not an exception in this global trend and an increase in mean BMI, prevalence of overweight, and obesity among children has also been observed [5, 6]. Young adult BMI is the sum of childhood BMI and pubertal BMI change. Due to the lack of cohorts with information on BMI both shortly before and shortly after puberty covering a large time span, the trend of pubertal BMI change has not previously been evaluated. To our knowledge, this is the first study investigating the trend for the new risk marker for cardiovascular disease, pubertal BMI change.

Adult obesity is an important risk factor behind cardiovascular and metabolic disease [17], and an elevated childhood BMI has also been shown to associate with an increased risk of cardiovascular diseases [2, 18]. Using a cohort with both prepubertal and postpubertal BMI available, we have demonstrated a robust association between pubertal BMI change and cardiovascular mortality, stroke, heart failure, acute coronary events, and type 2 diabetes. Moreover, the pubertal BMI change is associated with the expansion of the visceral fat [13], and with coronary atherosclerosis [12], independent of childhood BMI. The associations between pubertal BMI change and cardiovascular diseases are substantially more pronounced than the corresponding associations for childhood BMI and in addition, the associations for pubertal BMI change were independent of childhood BMI [9–11]. We propose that the secular trend of increasing pubertal BMI change might contribute more than the secular trend of increasing childhood BMI to the adverse cardiovascular health consequences associated with the ongoing obesity epidemic. A sustained trend of increased pubertal BMI change might even reverse the reduction of cardiovascular mortality that has been observed lately [19]. In this study, we show a secular trend of increasing pubertal BMI change. This is likely the result of the increasingly obesogenic environment and behavior change, including increased consumption of energy-dense foods and decreased physical activity.

Prevention of obesity during the childhood years is directed towards parents, while prevention of pubertal obesity needs to target the adolescents [15]. Evaluation of the timing of the increase in BMI during development will potentially have an impact on the framing of preventive measures and treatment of overweight and obesity.

A limitation of this study is that we only have data for boys. Moreover, data on pubertal Tanner stages was not available. The strengths of this study are the population-based nature of the study and the near-complete participation in the school healthcare program with repeated standardized measurements of height and weight in Sweden.

In conclusion, there is a secular trend of increased pubertal BMI change. As excessive BMI increase during puberty is a major risk factor for cardiovascular diseases, we propose that the secular trend of increasing pubertal BMI change might contribute more than the secular trend of increasing childhood BMI to the adverse cardiovascular health consequences associated with the ongoing obesity epidemic.

## Supplementary information

Supplemental material
