# Supplementary material for: A secular trend of increasing pubertal BMI change among Swedish adolescents
Source: Int J Obes (Lond). 2021 Nov 6;46(2):444–6. doi: 10.1038/s41366-021-01011-0 (PMC8794784; doi:10.1038/s41366-021-01011-0)
Supplement: Supplementary file 1 — Supplemental material [file 41366_2021_1011_MOESM1_ESM.docx]

# Supplemental material

# A secular trend of increasing pubertal BMI change among Swedish adolescents

Maria Bygdell, Claes Ohlsson, Jenny M Kindblom

**Supplemental methods**

Prepubertal childhood BMI and young adult BMI were calculated using all paired height and weight measurements during the period between 6.5 and 9.5 years of age for prepubertal childhood BMI, and in the period 17.5 to 22 years of age for young adult BMI. All measurements within these intervals were used to construct a linear regression model and the data for individual subjects were then adjusted on the slope of the regression to obtain BMI at 8 and 20 years of age, respectively. Pubertal BMI change was calculated as the difference between young adult BMI and childhood BMI. The time interval 8-20 years was chosen in order to avoid the confounding effect of ongoing puberty on BMI.

Childhood height was estimated with a model similar to childhood BMI. All height measurements between 6.5 and 9.5 years of age were used and age-adjusted with a linear regression model to age 8 years. For final height for individuals with information on height between 21 and 50 years of age in the Passport register, we used the mean of available heights for each individual (available for 79.9% of the study cohort). For adult height for individuals with no information on height after 21 years of age in the Passport register, we used height measurements from school healthcare and/or the Conscription register (20.1%). Height measurements in the interval 17.5 to 22 years of age were age-adjusted to 21 years of age. For individuals with several measurements in the interval, the mean of their age-adjusted heights was used.

**Supplemental Table 1**

Table 1. Descriptive statistics for the total cohort (n=3650). Childhood underweight, overweight, and obesity categorized according to the Centers for Disease Control and Prevention’s definitions at 13.8, 17.9, and 20.0 kg/m^2^ respectively. Young adult underweight, overweight, and obesity were defined as 18.5, 25-30, and >30 kg/m^2^, respectively.

| **Variable** | **Mean (SD)** | **n (%)** |
| --- | --- | --- |
| Childhood BMI (8 years, kg/m^2^) | 16.0 (1.7) |  |
| Underweight |  | 141 (3.9) |
| Overweight |  | 598 (16.4) |
| Obesity |  | 107 (2.9) |
| Young adult BMI (20 years, kg/m^2^) | 22.0 (3.1) |  |
| Underweight |  | 271 (7.4) |
| Overweight |  | 444 (12.2) |
| Obesity |  | 91 (2.5) |
| Pubertal BMI change (kg/m^2^) | 6.0 (2.3) |  |
| Childhood height (8 years, cm) | 130.0 (5.4) |  |
| Final height (cm) | 181.0 (6.6) |  |

BMI=Body Mass Index, SD=Standard Deviation
